# Revealing the Intersection: Scleroderma Renal Crisis Complicating Membranous Nephropathy

**DOI:** 10.7759/cureus.83327

**Published:** 2025-05-01

**Authors:** Ashar Uddin Kazi, Saifuddin Mohammad Kibria, Monowara Rahman Shipi, Rameen Ahmed, Rafid Mustafa

**Affiliations:** 1 Internal Medicine, United Lincolnshire Teaching Hospitals NHS Trust, Lincoln, GBR; 2 Medicine, Lincoln Medical School, University of Nottingham, Lincoln, GBR

**Keywords:** diffuse systemic sclerosis, primary membranous nephropathy, scleroderma clinical trials consortium (sctc), scleroderma renal crisis, united kingdom scleroderma study group (ukssg)

## Abstract

An 82-year-old woman with systemic sclerosis (anti-topoisomerase I (anti-Scl-70) positive) and a history of membranous nephropathy presented with a prolonged illness marked by worsening oedema, reduced urine output, and severe hypertension. Laboratory investigations revealed nephrotic-range proteinuria, acute kidney injury (AKI), and a marked decline in renal function. Initial treatment with diuretics and antihypertensive therapy yielded limited improvement. Renal biopsy demonstrated dual pathology: primary membranous nephropathy and acute vascular changes indicative of scleroderma renal crisis (SRC). Despite targeted therapy, the patient experienced flash pulmonary oedema, necessitating haemodialysis. This case highlights the complexities of diagnosing and managing overlapping renal pathologies in systemic sclerosis, underscoring the critical importance of early recognition and intervention in SRC.

## Introduction

Systemic sclerosis (SSc) is a chronic connective tissue disorder that leads to the gradual thickening and scarring of the skin and internal organs. One of its most critical renal complications, known as scleroderma renal crisis (SRC), is characterized by severe high blood pressure, acute kidney injury (AKI), and a significantly increased risk of mortality [1]. Recent reports have highlighted an increasing number of cases of renal crisis occurring without skin involvement. Diagnosing SRC is challenging when typical skin manifestations and autoantibodies are not present [2,3]. This case underscores the difficulties of diagnosing and managing overlapping renal pathologies in SSc, especially since our patient did not have typical risk factors, highlighting the critical importance of early recognition and intervention in SRC.

## Case presentation

An 82-year-old woman presented with a prolonged feeling of unwellness and progressively worsening leg swelling over the past 4-5 months, which has extended to her lower abdomen. She also complained of reduced urine output, though she denied any urinary symptoms such as frequency, urgency, dysuria, or abdominal pain. She experienced no respiratory symptoms, including shortness of breath, chest pain, orthopnoea, or paroxysmal nocturnal dyspnoea, and denied any history of dysphagia or chest pain. Additionally, she reported worsening pain in her hands and wrists but denied taking steroids or anti-inflammatory medications before her hospital admission. Upon admission, her vital signs showed a heart rate of 85 bpm, a respiratory rate of 29 breaths per minute, a significantly elevated blood pressure of 205/111 mmHg, a normal temperature of 36.6°C, and an oxygen saturation of 99% on room air. She also noted a history of hypotension, though her blood pressure had not been monitored recently.

The patient has a background of membranous nephropathy, diagnosed 40 years ago, which resolved spontaneously without the need for treatment. She also has SSc with anti-topoisomerase I (anti-Scl-70) positivity, primarily manifesting as Raynaud's phenomenon, for which she is not currently receiving any treatment. On physical examination, she had 3+ bilateral pitting oedema extending up to her pelvis. Auscultation of the chest revealed bilateral basal crepitations, though her jugular venous pressure was not elevated. Examination of her hands revealed sclerodactyly, with some dilated nailfold capillaries, but no digital ulcers were present. Other systemic examinations did not reveal any significant abnormalities. She had chronic hyponatraemia so her general practitioner has started her on sodium chloride table 600 mg twice daily (BD) and for oedema bumetanide 1 mg once daily (OD).

**Figure 1:**
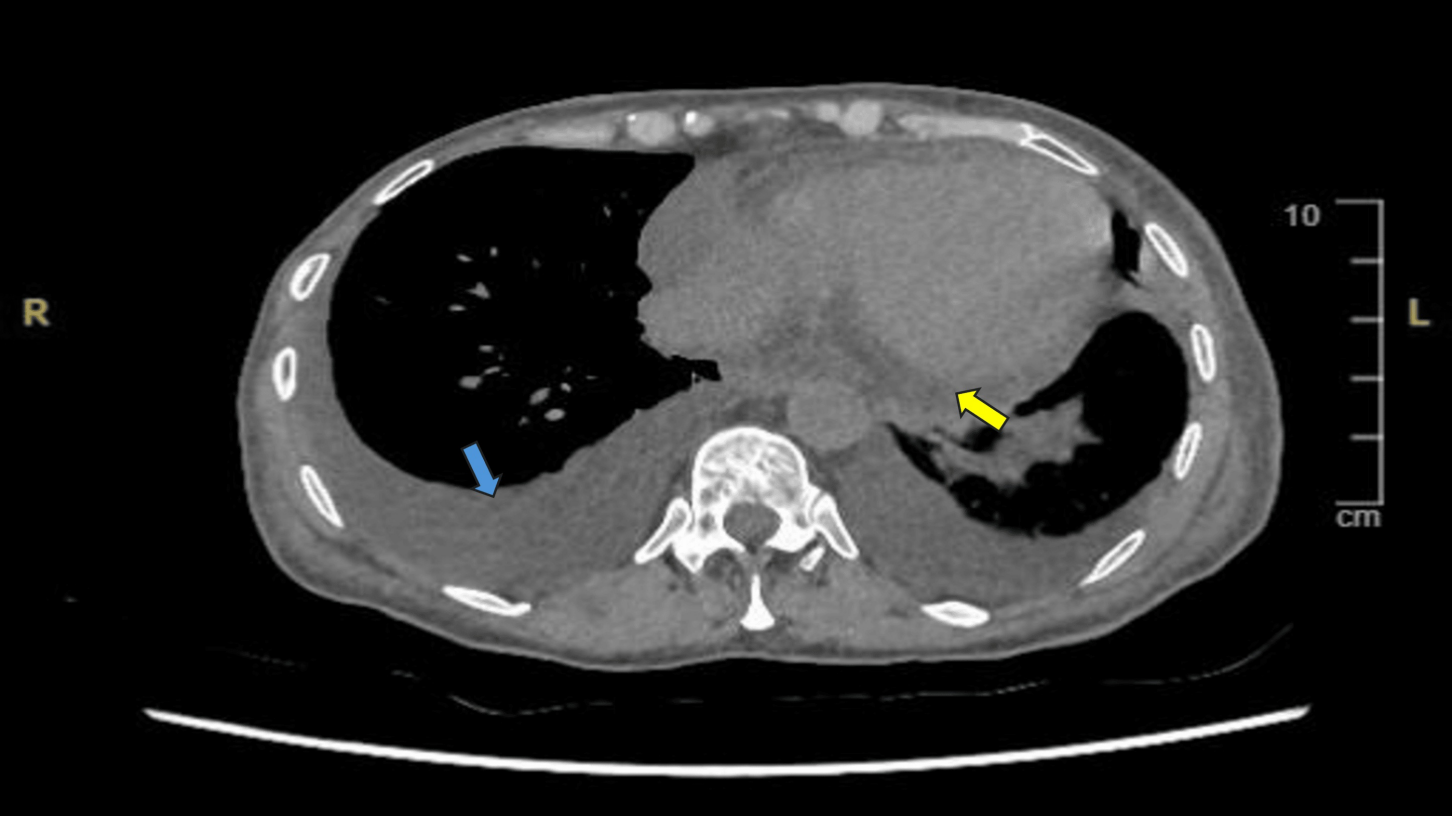
CT KUB of the patient (chest view) The blue arrow indicated pleural effusion. The yellow arrow indicated pericardial effusion. CT KUB: computed tomography of the kidneys, ureters, and bladder

Upon admission, the patient's blood test results revealed a sodium level of 130 mmol/L, a creatinine level of 144 µmol/L, an albumin level of 22 g/L, and an estimated glomerular filtration rate (eGFR) of 29 mL/min (Table 1), a significant decline from her baseline eGFR of 85 mL/min. Her urine dipstick test showed protein (+), blood (+++), with no leukocytes or nitrites, and a urine pH of 6.0. A computed tomography of the kidneys, ureters, and bladder (CT KUB) revealed no renal or ureteric tract calculi or hydronephrosis. However, there were diffuse oedema in the soft tissues of the abdominal and chest walls, bilateral moderate pleural effusions, moderate pericardial effusion, non-specific oedema of the ascending colon, and free fluid in the pelvis (Figure 1). A later urinary sample showed an albumin level of >400 mg/L, a urinary protein of 1698 mg/L, and a urine protein-to-creatinine ratio of 291 mg/mmol, indicating nephrotic-range proteinuria.

**Table 1:** In-patient investigations Sodium: 133-146 mmol/L; potassium: 3.5-5.3 mmol/L; urea: 2.5-7.8 mmol/L; creatinine: 59-104 µmol/L; GFR: 90-200 mL/min

Day	Sodium (mmol/L)	Potassium (mmol/L)	Urea (mmol/L)	Creatinine (µmol/L)	GFR (mL/min)
On admission	130	3.5	10.6	144	29
3	126	3.4	13.9	167	24
4	129	2.8	14.4	189	21
5	129	2.7	16.0	200	20
6	129	3.7	19.7	238	16
7	130	4.6	26.2	310	12
8	128	4.1	29.2	343	10
9	127	4.6	31.2	398	9
10	127	4.6	35.1	440	8
11	126	4.7	37.1	491	7
12	125	4.3	37.0	501	6
13	126	4.0	34.0	475	7
14	127	4.1	27.0	450	7
15	127	4.3	20.8	413	8
16	128	3.6	14.2	375	9
17	125	4.1	16.9	457	7
18	123	3.9	20.7	536	6
19	127	4.1	14.3	444	7
20	127	3.7	16.2	511	6
21	125	3.7	19.7	582	5
22	128	3.6	13.7	477	7
23	127	3.5	17.4	567	6
24	126	4.7	23.4	680	4
25	125	5.2	27.4	721	4
27	129	3.8	21.9	625	5
28	130	3.7	14.6	451	7
29	130	3.4	13.7	486	7
30	133	3.9	9.0	392	9
32	130	3.6	14.1	600	5
33	130	3.7	9.3	451	7
34	131	3.5	12.6	557	6
35	132	3.3	8.7	435	8
36	130	3.4	9.9	463	7
37	132	3.3	7.6	386	9
38	130	3.7	11.5	488	7
39	129	3.5	14.5	590	5
40	130	4.1	9.4	458	7
41	135	4.6	14.3	579	

Initially, the patient was started on intravenous furosemide 40 mg BD, with fluid restriction to 1.5 L/day, and her fluid balance was closely monitored through strict input and output measurements. The renal team reviewed her case and suspected a relapse of membranous nephropathy. While SRC was considered due to her elevated blood pressure, it was deemed less likely as this condition typically does not present with heavy proteinuria. The renal team advised an urgent transfer to the renal unit for further evaluation, as a biopsy may be necessary. To manage her blood pressure, amlodipine 5 mg OD was initiated, along with captopril 6.25 mg BD for its antiproteinuric effect.

She was continued with IV diuresis and was planned for biopsy after optimising blood pressure. Immunology screening was done (Table 2).

**Table 2:** Autoantibody investigations Ab: antibody; SS-B: Sjogren's syndrome antigen B; SS-A: Sjogren's syndrome-related antigen A autoantibodies; Ig: immunoglobulin; Sm: anti-Smith; ANA: anti-nuclear antibodies; ENA: extractable nuclear antigen; Scl-70: topoisomerase I; U1 RNP: U1 ribonucleoprotein

Test	Result	Range/unit
ANA	Positive IgG 1:320 (positive)	-
Double-stranded DNA Ab	<9.8	0-27 IU/mL
ENA Ab screen	Positive	-
JO-1 Ab	Positive	-
Scl-70 Ab (anti-topoisomerase)	Positive	-
La Ab (SS-B)	Positive	-
Ro-52 Ab	Positive	-
Ro-Ab (SS-A)	Positive	-
Sm Ab	Negative	-
U1 RNP Ab	Negative	-
Myeloperoxidase Ab	<3.2	0-20 U
Proteinase-3 Ab	<2.3	0-20 U
Rheumatoid factor	60	0-14 IU/mL
Glomerular membrane basement Ab	<2.9	0-20 IU
Serum electrophoresis/Igs	Unremarkable pattern, no evidence of a monoclonal band	-
IgG	16.06	7-16 g/L
IgA	1.99	0.7-4.0 g/L
IgM	1.66	0.4-2.3 g/L
Serum kappa	97.2	3.3-19.4 mg/L
Serum lambda	72.2	5.7-26.3 mg/L
Serum kappa/lambda ratio	1.35	0.26-1.65 ratio
Phospholipase A2 receptor Ab	7	0-13 RU/mL

**Figure 2:**
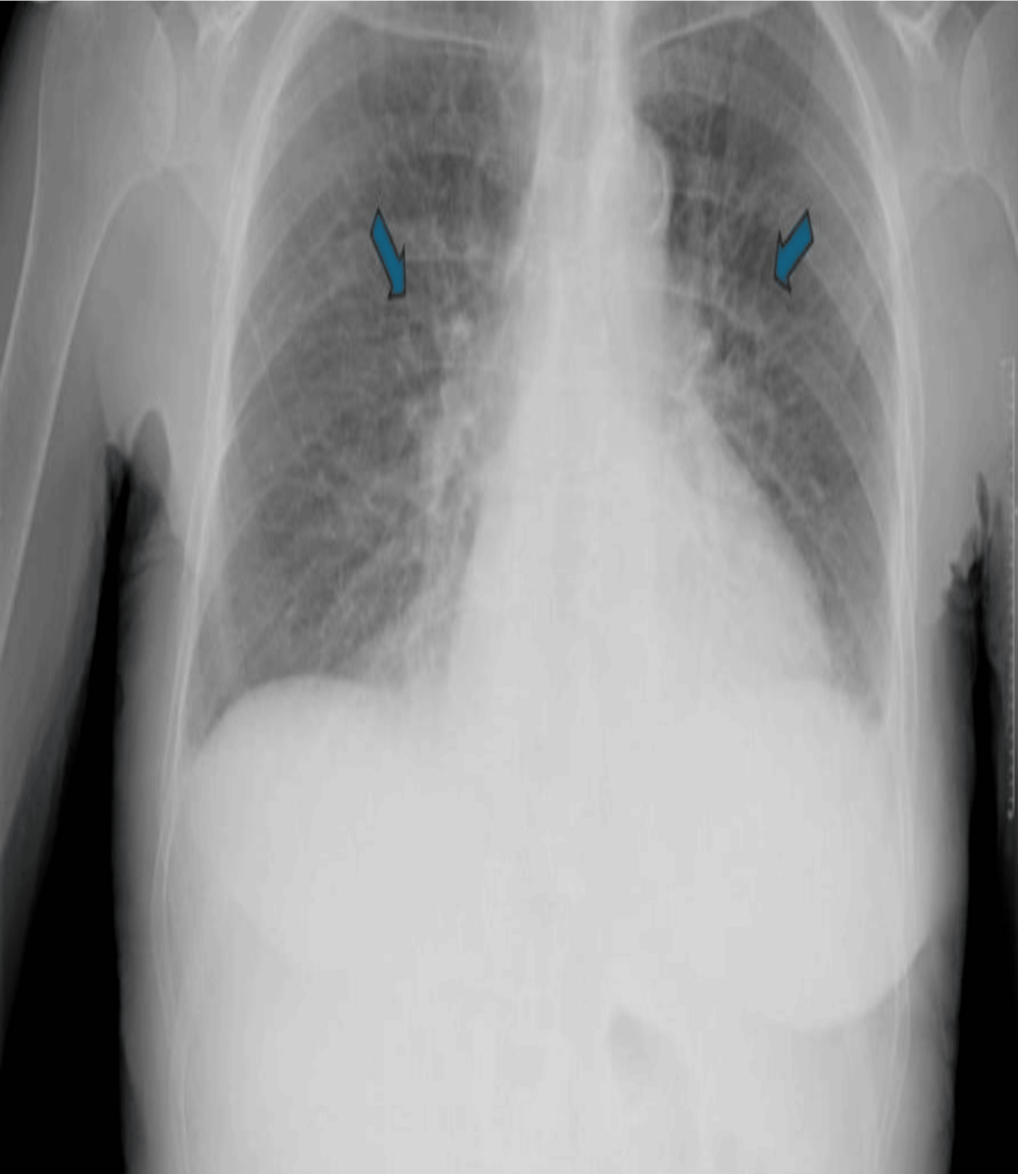
Chest X-ray during hospital stay The arrows indicate pulmonary congestion.

The patient's renal function was not improving, and she remained fluid-overloaded, exhibiting features of orthopnoea, paroxysmal nocturnal dyspnoea, and raised jugular venous pressure. Consequently, her furosemide dose was increased to 80 mg BD, and captopril was suspended. Despite these measures, her renal function continued to deteriorate, and she experienced episodes of flash pulmonary oedema (Figure 2), necessitating the initiation of haemodialysis. Once she was offloaded and her blood pressure normalized, a renal biopsy was performed.

The biopsy revealed a core of renal cortex and medulla containing approximately 14 glomeruli in a single plane of section, one of which was globally sclerosed. The glomeruli displayed a solid, bloodless appearance with mostly closed capillary loops, and where lumina were visible, they appeared punched out with thickened capillary walls. There was no mesangial matrix expansion or hypercellularity, and the capillary loops appeared wrinkled and collapsed on structural stains. Sub-epithelial spikes, double contours, and endocapillary hypercellularity were not present. One glomerulus showed a segmental sclerotic lesion.

Approximately 40% of the sampled cortex was affected by interstitial fibrosis and tubular atrophy. Non-atrophic tubules exhibited acute tubular injury with luminal dilatation and the attenuation of apical cytoplasm. No evidence of tubulointerstitial nephritis was observed. Multiple extraglomerular muscular-walled arteries of varying calibres were sampled, all showing abnormalities, including acute injury characterized by red cell fragmentation within the vessel wall, endothelial swelling, and narrowed or occluded lumina. Some vessels also displayed myointimal hyperplasia or "onion skinning," along with mild fibrointimal thickening and elastin reduplication. These findings are indicative of acute vascular injury with superimposed chronic changes.

**Figure 3:**
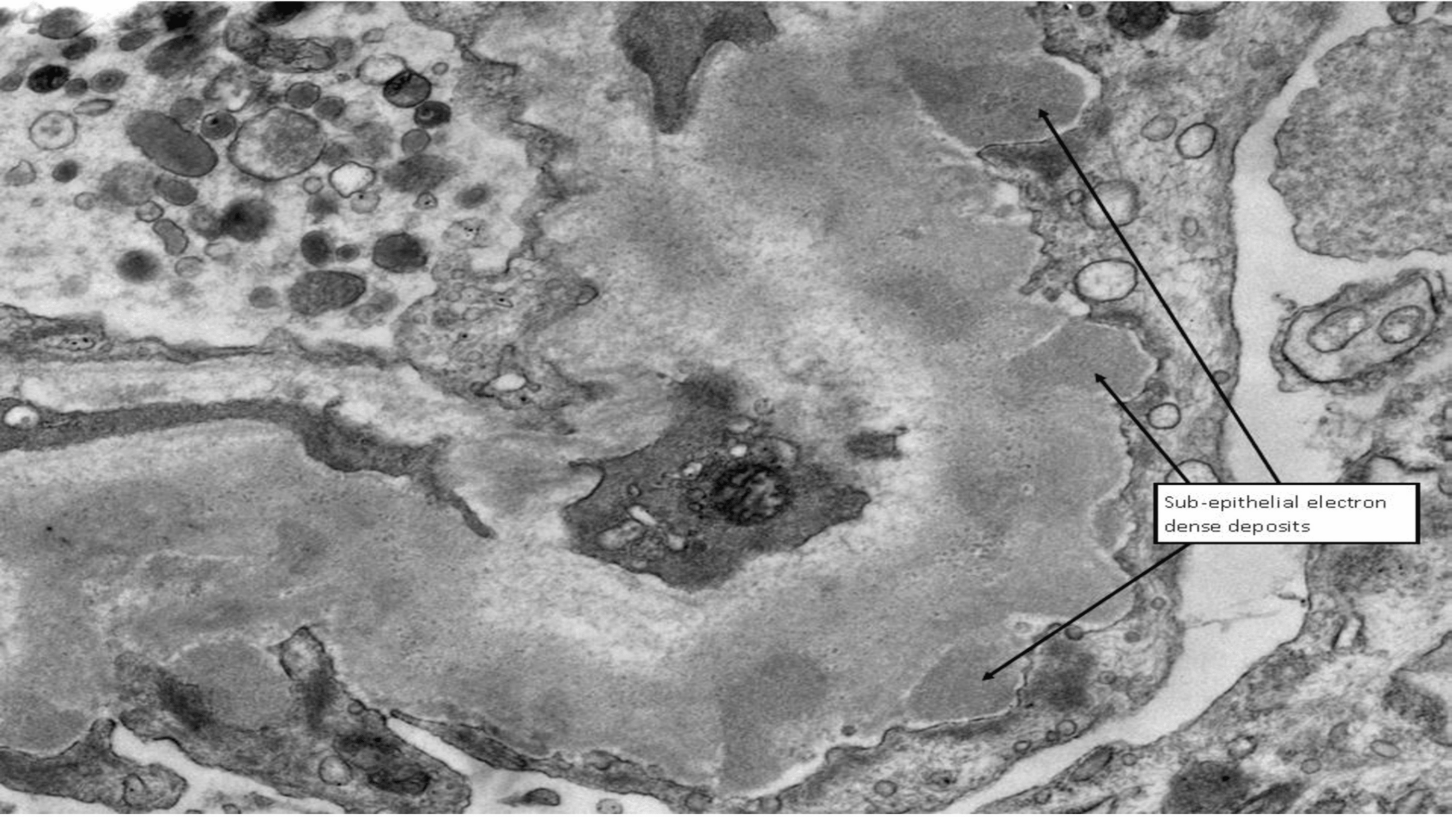
Electron microscopy image of the renal biopsy The arrows in this electron microscopy image are pointing to sub-epithelial electron-dense deposits, which are located between the podocyte foot processes and the glomerular basement membrane.

**Figure 4:**
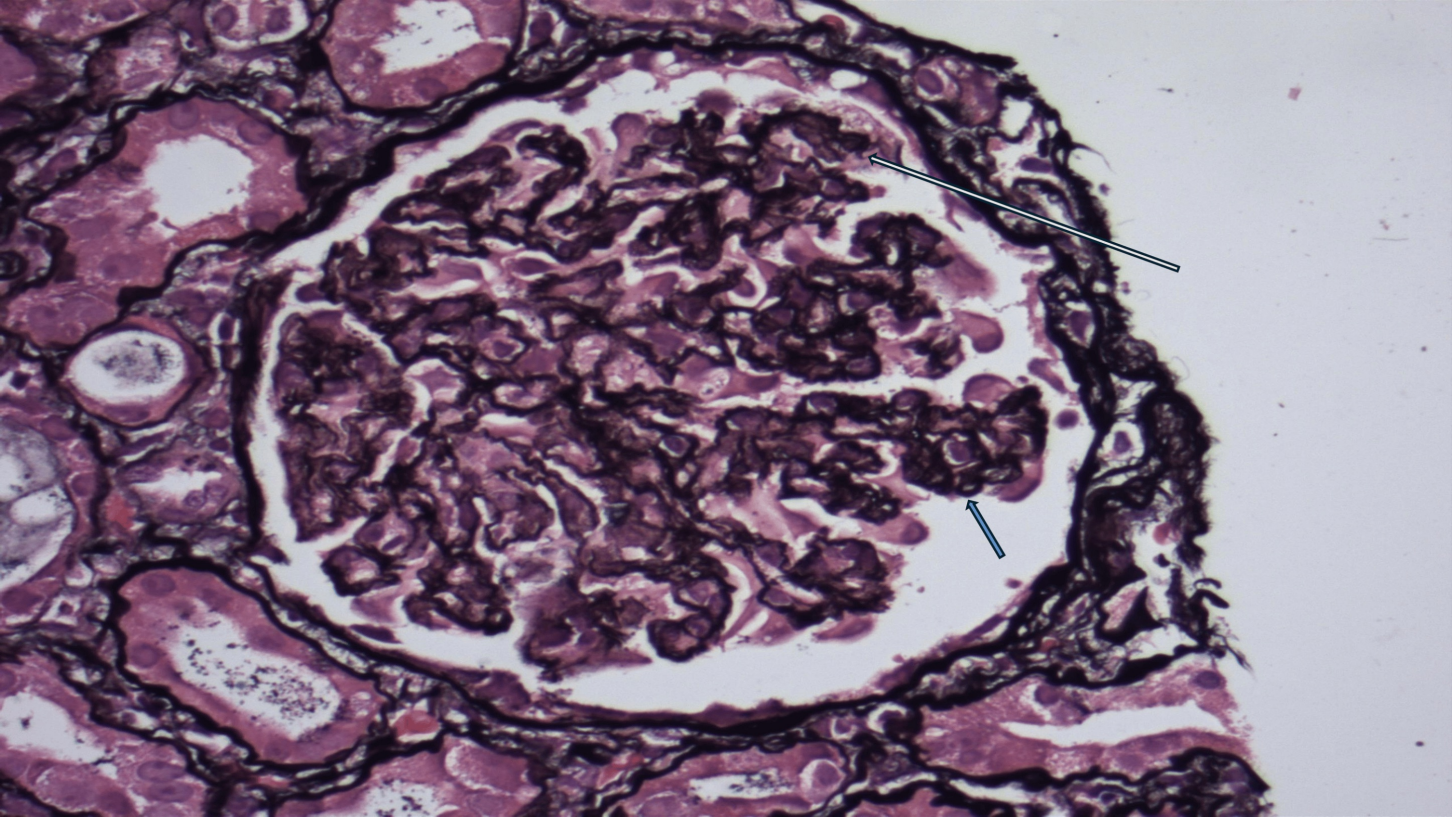
Jones methenamine silver stain of the renal biopsy The white arrow shows wrinkled and collapsed capillary loops. The blue arrow indicates areas with no visible spikes along the glomerular basement membrane.

**Figure 5:**
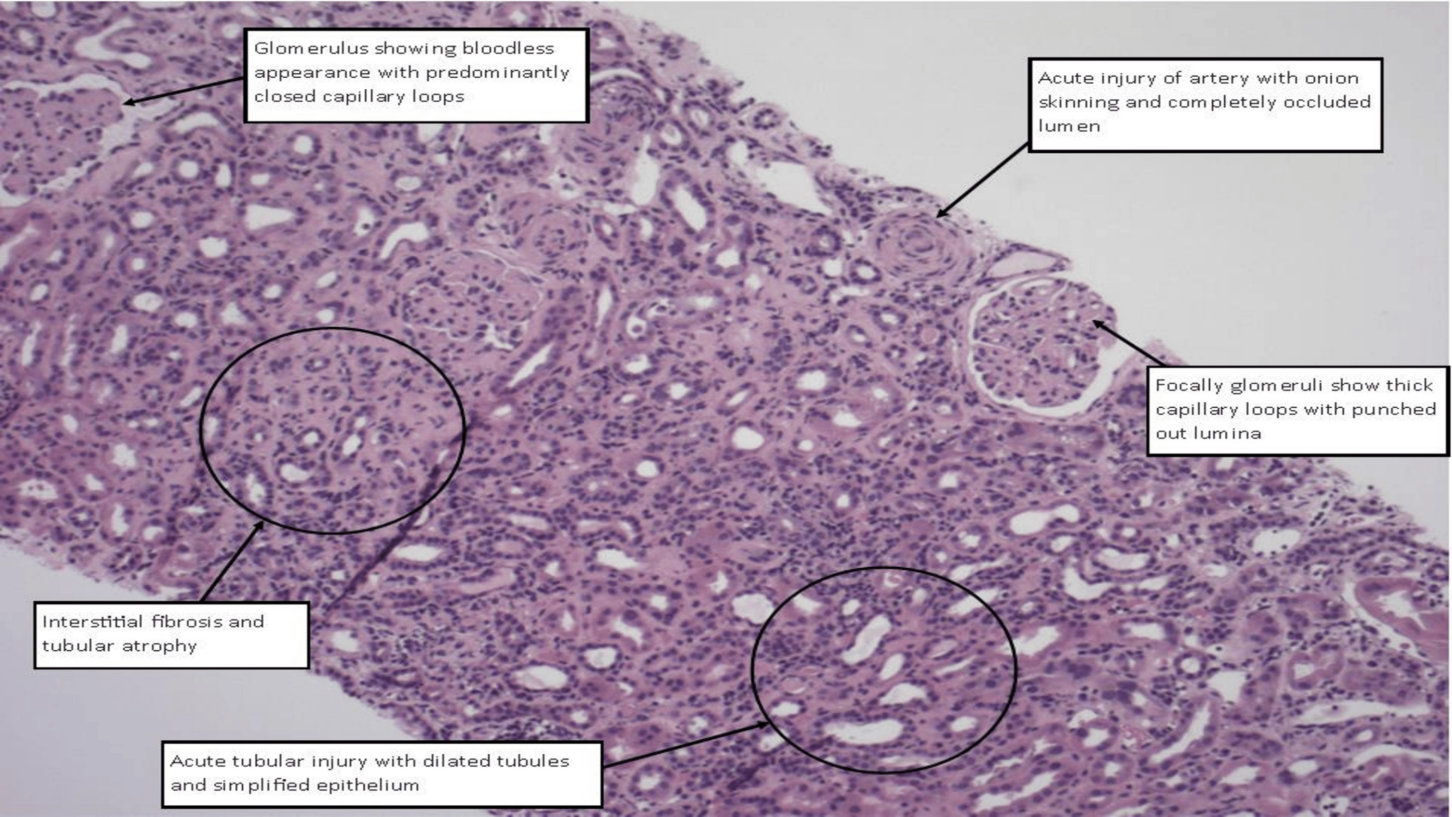
Hematoxylin and eosin-stained section of the renal biopsy The glomerulus (top left) shows a bloodless appearance with predominantly closed capillary loops. An artery (top right) exhibits acute injury with onion skinning and a completely occluded lumen. Some glomeruli (mid-right) show thickened capillary loops with punched-out lumina. The interstitium (left center) demonstrates interstitial fibrosis and tubular atrophy. The tubules (bottom right) display acute tubular injury with dilatation and simplified epithelium.

Immunofluorescence analysis of tissue containing four glomeruli showed granular staining within capillary loops with relative sparing of the mesangium on immunoglobulin G (IgG) (++), IgG4 (++), and C3 (++). Staining for IgA, IgM, and C1q was negative, and no light chain restriction was noted. These findings, in conjunction with light and electron microscopy, were consistent with membranous nephropathy. Phospholipase A2 receptor (PLA2R) immunoperoxidase staining confirmed primary membranous nephropathy. However, the biopsy also showed features of acute endothelial injury, such as acute vascular changes and the solid, bloodless appearance of the glomeruli, which were indicative of an SRC. While this vascular injury is most likely attributable to the patient's known scleroderma, it could not be determined histologically and was unrelated to the co-existing membranous glomerulonephritis (Figure 3, Figure 4, and Figure 5). 

She was continued on thrice-weekly dialysis, for which a tunnelled line was inserted. At discharge, she remained dialysis-dependent and was scheduled for haemodialysis sessions on Tuesday, Thursday, and Saturday evenings, with a target weight of 50 kg. She was discharged with a prescription for enalapril 2.5 mg OD and darbepoetin alfa 40 mcg/0.4 mL injection to be administered once weekly.

The patient was discharged with the prescribed medication and follow-up plan. Unfortunately, she did not attend the scheduled follow-up appointment.

## Discussion

SSc can be categorized based on the extent of skin involvement into three subtypes: limited cutaneous systemic sclerosis (lcSSc), diffuse cutaneous systemic sclerosis (dcSSc), and systemic sclerosis sine scleroderma (ssSSc). In lcSSc, skin fibrosis usually affects the acral regions, including the face and distal extremities. It has the most favourable prognosis, with a 10-year survival rate exceeding 90%. On the other hand, dcSSc has a more severe prognosis due to the rapid advancement of skin thickening and internal organ involvement. The 10-year survival rate for dcSSc ranges from 65% to 82% [2].

Renal involvement in ssSSc can manifest in two distinct forms: SRC and chronic form. SRC is marked by the sudden onset of hypertension and the rapid development of AKI [2]. It has been reported that SRC develops in 4.2% of patients with dcSSc and in just 1.1% of patients with lcSSc [1]. The development of SRC is linked to damage to the vascular endothelial cells of the interlobular and arcuate arteries in the kidneys. This damage results in intimal proliferation, which diminishes renal perfusion and leads to hyperplasia of the juxtaglomerular apparatus. Consequently, there is an increase in renin secretion, which contributes to the onset of acute hypertension and acute renal dysfunction [1].

In 2015, the Scleroderma Clinical Trials Consortium (SCTC) conducted a scoping review and consensus study to develop widely applicable classification criteria for SRC [4,5]. The group identified a core set of variables associated with SRC. These variables are currently being evaluated in real-world patients through the International Scleroderma Renal Crisis Survey II (ISRCS II) to assess their accuracy and specificity. The key parameters under investigation include AKI, high blood pressure, microangiopathic haemolytic anaemia (MAHA), thrombocytopenia (low platelet count), target organ damage, and renal tissue pathology [4,5] (Table 3).

**Table 3:** SRC classification criteria as defined by the SCTC Working Group AKI: acute kidney injury; SRC: scleroderma renal crisis; LDH: lactate dehydrogenase; ECG: electrocardiogram; MAHA: microangiopathic haemolytic anaemia; SCTC: Scleroderma Clinical Trials Consortium

Domain	
Blood pressure	An acute rise in blood pressure is characterized by any of the following:
	Systolic blood pressure of 140 mmHg or higher
	Diastolic blood pressure of 90 mmHg or higher
	An increase of 30 mmHg or more in systolic blood pressure from baseline
	An increase of 20 mmHg or more in diastolic blood pressure from baseline. Blood pressure should be measured twice, with at least a 5-minute interval between readings. If the measurements are inconsistent, additional readings should be taken until two consistent results are obtained
Kidney injury	AKI is characterized by any of the following criteria:
	An increase in serum creatinine of ≥26.5 μmol/L (≥0.3 mg/dL) within a 48-hour timeframe
	A rise in serum creatinine to ≥1.5 times the baseline, known or presumed to have occurred in the past 7 days
	A urine output of less than 0.5 mL/kg/h for at least 6 hours
MAHA and thrombocytopenia	New or worsening anaemia not linked to other causes
	Detection of schistocytes or other red blood cell fragments on a blood smear
	Thrombocytopenia defined as platelet counts ≤100,000 platelets/mm³, confirmed by a manual smear
	Laboratory evidence of haemolysis, including elevated LDH, reticulocytosis, and/or low or absent haptoglobin
	A negative direct antiglobulin test
Target organ dysfunction	Hypertensive retinopathy: identified by haemorrhages, hard and soft (cotton wool) exudates, and/or disc oedema, not due to other causes, and confirmed by an ophthalmologist
	Hypertensive encephalopathy: characterized by headache, altered mental status, seizures, visual disturbances, or other neurological signs not due to other causes
	Acute heart failure: marked by symptoms such as breathlessness, ankle swelling, and fatigue, along with signs like elevated jugular venous pressure and pulmonary crackles
	Acute pericarditis: diagnosed with at least two of the following: (1) chest pain, (2) pericardial rub, (3) new widespread ST-segment elevation or PR-segment depression on ECG, and (4) new or worsening pericardial effusion seen on echocardiography
Renal histopathology	Histopathologic features observed in a kidney biopsy that align with SRC may include the following:
	Alterations are more prominent in the small vessels, particularly the arcuate and interlobular arteries, with glomerular changes being less significant
	Thrombotic microangiopathy-related glomerular changes may be present, with acute features like fibrin thrombi, endothelial swelling, fragmented red blood cells, and mesangiolysis. Chronic changes may show double contouring of the glomerular basement membrane
	Non-specific ischemic alterations may include folding of the glomerular basement membrane, along with possible segmental or global sclerosis of the glomeruli
	Early vascular changes may feature the deposition of myxoid material in the intimal layer, along with thrombosis, fibrinoid necrosis, and the presence of fragmented red blood cells, which can sometimes lead to cortical necrosis
	The constriction and blockage of the vascular lumen result in glomerular ischemia. While infrequent (around 10%), hyperplasia of the juxtaglomerular apparatus may be noted
	In the later stages, changes include intimal thickening and proliferation, leading to the characteristic "onion-skin" lesions in the vessels, as well as glomerulosclerosis and interstitial fibrosis
	Non-specific tubular alterations may occur, including acute tubular injury in the early stages and later developments such as interstitial fibrosis and tubular atrophy. As these findings are not exclusive to SRC, the diagnosis must be confirmed with appropriate clinical and serological data

SRC is linked to several risk factors. The highest risk is observed in patients with early dcSSc and proximal skin thickening. Anti-ribonucleic acid (anti-RNA) polymerase III antibodies, frequently associated with dcSSc, have a strong correlation with SRC, with up to 50% of antibody-positive patients developing the condition [6]. Similarly, anti-topoisomerase antibodies (ATA), another marker of dcSSc, are implicated in about 10% of cases [7]. In contrast, SRC is rarely seen in lcSSc with anti-centromere antibodies (ACA) [8].

Genetic factors such as human leukocyte antigen (HLA)-DRB1*1407 and HLA-DRB1*1304 independently increase SRC risk [9], and potential associations between anti-RNA polymerase III antibodies and endothelin receptor type A (EDNRA) polymorphisms have been suggested, though their functional significance remains uncertain [10].

SRC can develop in the early stages of SSc, even before diffuse cutaneous involvement occurs. Early indicators include tendon friction rubs, polyarthritis, swollen hands, and carpal tunnel syndrome, which may later progress to skin thickening [11]. Additional risk factors are dcSSc, anaemia, pericardial effusion, heart failure, rapid skin thickening, large joint contractures, cardiac enlargement, proteinuria [4], and corticosteroid use, particularly at doses higher than 15 mg/day [12]. The risk is especially high with high-dose glucocorticoids, highlighting the need for careful medication management.

Most patients with SRC have anti-nuclear antibodies (ANA), with anti-RNA polymerase (types I and III) and anti-Scl-70 antibodies strongly linked to SRC in dcSSc [13]. In contrast, anticentromere antibodies, typically seen in lcSSc, are rarely associated with SRC, and the connection between anti-U3 ribonucleoprotein (anti-U3 RNP) antibodies and SRC is inconsistent [13]. Additionally, up to 50% of SRC patients develop microangiopathic haemolytic anaemia, characterized by sudden anaemia, schistocytes, thrombocytopenia, elevated lactate dehydrogenase (LDH), and low haptoglobin, often indicating thrombotic microangiopathy [13].

SRC is a critical condition that can arise during the progression of SSc, marked by malignant hypertension and the rapid deterioration of renal function, but patients may also experience symptoms like headache, blurred vision, fatigue, or dyspnoea. Severe cases may involve seizures or pericardial involvement. Even if blood pressure appears normal, a significant increase from an individual's baseline can suggest SRC, with around 10% of cases being normotensive. Cardiac complications, often exacerbated by underlying cardiac scleroderma, are common and typically improve with tight blood pressure control, targeting the activation of the renin-angiotensin-aldosterone (RAA) axis [4].

The United Kingdom Scleroderma Study Group (UKSSG) has established diagnostic criteria for SRC (Table 4) [14].

**Table 4:** UKSSG diagnostic criteria for SRC 2016 Reproduced with permission from Lynch et al. [14]. UKSSG: United Kingdom Scleroderma Study Group; MAHA: microangiopathic haemolytic anaemia; SRC: scleroderma renal crisis

Essential diagnostic criteria
New-onset blood pressure >150/85 mmHg, measured at least twice within 24 hours
Systolic blood pressure increases by ≥20 mmHg from the individual's usual baseline
Acute kidney injury of stage 1 or higher, indicated by a >50% rise in serum creatinine from the stable baseline or an absolute increase of 26.5 µmol/L
Desirable supporting evidence
MAHA on blood smear, thrombocytopenia, and other biochemical indicators of haemolysis
Retinal examination showing signs consistent with accelerated hypertension
Microscopic haematuria on urine dipstick or presence of red blood cells in urine microscopy
Oliguria or anuria
Renal biopsy revealing typical features of SRC, such as onion skin proliferation in intrarenal arteries and arterioles, fibrinoid necrosis, and glomerular shrinkage
Flash pulmonary oedema

Angiotensin-converting enzyme (ACE) inhibitors are the primary choice of antihypertensive therapy for managing SRC due to their demonstrated benefits. Research has shown that these medications improve survival rates, help preserve kidney function, and contribute to better blood pressure regulation [15], with captopril frequently highlighted in studies [2]. After starting an ACE inhibitor, monitoring serum creatinine levels is crucial to identify any adverse changes in kidney function. While angiotensin II receptor blockers (ARBs) might offer similar benefits, their effectiveness in SRC has not been thoroughly studied [2]. Other treatment approaches include haemodialysis, which should be utilized when indicated to address complications such as fluid overload or electrolyte imbalances. Plasma exchange therapy, when combined with ACE inhibitors, has shown potential benefits, including a higher one-year renal survival rate compared to using ACE inhibitors alone [16]. On the other hand, corticosteroids should be avoided because they can worsen hypertension through salt and water retention, and studies have linked them to an elevated risk of SRC [17].

This case highlights several critical learning points related to the diagnosis and management of SRC in the context of SSc. First, the importance of recognizing the overlap of renal pathologies, especially in patients with established autoimmune conditions, is emphasized. SRC, often characterized by sudden hypertension and AKI, can present without typical skin changes, making early identification difficult. Renal biopsy findings, such as endothelial injury and glomerular changes, are crucial for distinguishing SRC from other renal pathologies, such as membranous nephropathy. Additionally, the patient's presentation reinforces the need for close monitoring of blood pressure and renal function, particularly in elderly patients with SSc who may have underlying kidney issues.

This case underscores the complexities of managing SSc with overlapping renal complications, particularly SRC. Early recognition and intervention are essential to prevent irreversible kidney damage and systemic complications. In patients with SSc, SRC should be considered even in the absence of classic cutaneous manifestations, especially when hypertension and AKI are present. The patient's management involved a combination of diuretics, ACE inhibitors, and eventual dialysis, which highlights the importance of a multidisciplinary approach. Despite targeted treatment, the patient's continued dialysis dependence and the need for close follow-up underline the severity of this renal crisis and the necessity for prompt, aggressive intervention.

## Conclusions

This case demonstrates the challenges in diagnosing and managing SRC in a patient with SSc, particularly when typical skin manifestations are absent. The dual pathology of primary membranous nephropathy and SRC underscores the complexity of renal involvement in SSc. Early recognition of SRC is crucial to initiate appropriate therapy, including ACE inhibitors, and prevent further renal deterioration.

However, this case also highlights the diagnostic uncertainty that can arise when overlapping autoimmune conditions complicate the clinical picture. Alternative causes of AKI, such as hypertensive emergency secondary to nephrotic syndrome, must be considered. Careful clinical vigilance and a broad differential diagnosis are essential in evaluating such complex presentations, particularly in elderly patients with underlying autoimmune diseases.

Despite intensive management, the patient's ongoing dialysis dependence underscores the severe nature of SRC and the necessity for early intervention to improve outcomes in high-risk cases.

## References

[1] Shimizu T, Iwamoto N, Okamoto M (2019). Scleroderma renal crisis complicated with thrombotic microangiopathy triggered by influenza B virus infection. Intern Med.

[2] Hansrivijit P, Omeonu KF, Lawal HO, Gangireddy M, Gadhiya KP, Dhatt RS (2020). A 45-year-old man with scleroderma renal crisis associated with a history of systemic sclerosis sine scleroderma. Am J Case Rep.

[3] Zwettler U, Andrassy K, Waldherr R, Ritz E (1993). Scleroderma renal crisis as a presenting feature in the absence of skin involvement. Am J Kidney Dis.

[4] Cole A, Ong VH, Denton CP (2023). Renal disease and systemic sclerosis: an update on scleroderma renal crisis. Clin Rev Allergy Immunol.

[5] Butler EA, Baron M, Fogo AB (2019). Generation of a core set of items to develop classification criteria for scleroderma renal crisis using consensus methodology. Arthritis Rheumatol.

[6] Sobanski V, Dauchet L, Lefèvre G (2014). Prevalence of anti-RNA polymerase III antibodies in systemic sclerosis: new data from a French cohort and a systematic review and meta-analysis. Arthritis Rheumatol.

[7] Bunn CC, Denton CP, Shi-Wen X, Knight C, Black CM (1998). Anti-RNA polymerases and other autoantibody specificities in systemic sclerosis. Br J Rheumatol.

[8] Nihtyanova SI, Sari A, Harvey JC (2020). Using autoantibodies and cutaneous subset to develop outcome-based disease classification in systemic sclerosis. Arthritis Rheumatol.

[9] Nguyen B, Mayes MD, Arnett FC (2011). HLA-DRB1*0407 and *1304 are risk factors for scleroderma renal crisis. Arthritis Rheum.

[10] Fonseca C, Renzoni E, Sestini P (2006). Endothelin axis polymorphisms in patients with scleroderma. Arthritis Rheum.

[11] Avouac J, Walker UA, Hachulla E (2016). Joint and tendon involvement predict disease progression in systemic sclerosis: a EUSTAR prospective study. Ann Rheum Dis.

[12] Steen VD, Medsger TA Jr (1998). Case-control study of corticosteroids and other drugs that either precipitate or protect from the development of scleroderma renal crisis. Arthritis Rheum.

[13] Batal I, Domsic RT, Medsger TA, Bastacky S (2010). Scleroderma renal crisis: a pathology perspective. Int J Rheumatol.

[14] Lynch BM, Stern EP, Ong V, Harber M, Burns A, Denton CP (2016). UK Scleroderma Study Group (UKSSG) guidelines on the diagnosis and management of scleroderma renal crisis. Clin Exp Rheumatol.

[15] Penn H, Howie AJ, Kingdon EJ (2007). Scleroderma renal crisis: patient characteristics and long-term outcomes. QJM.

[16] Cozzi F, Marson P, Cardarelli S (2012). Prognosis of scleroderma renal crisis: a long-term observational study. Nephrol Dial Transplant.

[17] Bose N, Chiesa-Vottero A, Chatterjee S (2015). Scleroderma renal crisis. Semin Arthritis Rheum.

